# Estimating local need for mental healthcare to inform fair resource allocation in the NHS in England: cross-sectional analysis of national administrative data linked at person level

**DOI:** 10.1192/bjp.2019.185

**Published:** 2020-06

**Authors:** Laura Anselmi, Anna Everton, Robert Shaw, Wataru Suzuki, Jeremy Burrows, Richard Weir, Roman Tatarek-Gintowt, Matt Sutton, Stephen Lorrimer

**Affiliations:** 1 Research Fellow, Health Organisation, Policy and Economics, University of Manchester, UK; 2 Senior Analytical Lead, Analysis and Insight for Finance, NHS England, UK; 3 Lead Analysis (forecasting), Analytical Insight Resource Unit, NHS England, UK; 4 Senior Manager, Operations & Information Directorate, NHS England, UK; 5 Senior Analytical Manager, Analysis and Insight for Finance, NHS England, UK; 6 Analysis and Insight for Finance, NHS England, UK; 7 Analyst, Analysis and Insight for Finance, NHS England, UK; 8 Professor of Health Economics, Health Organisation, Policy and Economics, University of Manchester, UK; and Professorial Research Fellow, Melbourne Institute for Applied Economic and Social Research, University of Melbourne, Australia; 9 Head of Analysis and Insight for Finance, NHS England, UK

**Keywords:** Mental health, weighted capitation, need, cost

## Abstract

**Background:**

Equitable access to mental healthcare is a priority for many countries. The National Health Service in England uses a weighted capitation formula to ensure that the geographical distribution of resources reflects need.

**Aims:**

To produce a revised formula for estimating local need for secondary mental health, learning disability (intellectual disability) and psychological therapies services for adults in England.

**Method:**

We used demographic records for 43 751 535 adults registered with a primary care practitioner in England linked with service use, ethnicity, physical health diagnoses and type of household, from multiple data-sets. Using linear regression, we estimated the individual cost of care in 2015 as a function of individual- and area-level need and supply variables in 2013 and 2014. We sterilised the effects of the supply variables to obtain individual-need estimates. We aggregated these by general practitioner practice, age and gender to derive weights for the national capitation formula.

**Results:**

Higher costs were associated with: being 30–50 years old, compared with 20–24; being Irish, Black African, Black Caribbean or of mixed ethnicity, compared with White British; having been admitted for specific physical health conditions, including drug poisoning; living alone, in a care home or in a communal environment; and living in areas with a higher percentage of out-of-work benefit recipients and higher prevalence of severe mental illness. Longer distance from a provider was associated with lower cost.

**Conclusions:**

The resulting needs weights were higher in more deprived areas and informed the distribution of some 12% (£9 bn in 2019/20) of the health budget allocated to local organisations for 2019/20 to 2023/24.

Mental illness has been recognised as a worldwide problem, but the gap between provision and need for treatment is estimated to be between 35% and 50% in high-income countries. Increasing the provision of mental healthcare and ensuring that it is distributed equitably are now priorities for many countries.^1^

In England, 5.7% of the adult population reported a long-term mental health problem and 13.7% reported depression and/or anxiety in 2016.^2^ The Health and Social Care Act 2012 made NHS England responsible for delivering ‘parity of esteem’ between physical and mental health. The Government has committed to increasing funding by up to 10% (£1 bn per year by 2020/21^3^) to support its future strategy.^4^ The overall budget for mental health services was £12 bn in 2017, of which 85% was distributed through 207 local health organisations (clinical commissioning groups, CCGs). More recently, NHS England's Long Term Plan^5^ commits to a growing share of the National Health Service (NHS) budget for mental health, worth at least £2.3 bn per year in real terms by 2023/24.

NHS England allocates resources to CCGs on the basis of target shares of national resources derived from weighted capitation formulae. There are separate formulae for different funding streams, including general and acute, maternity, mental health, prescribing and primary care services. Specialised services are commissioned centrally.^6^ The need-based shares derived from each formula are then aggregated to determine each CCG's fair shares of the total budget.^7,8^ CCGs are then responsible for commissioning services. These include psychological therapies, as well as community, acute and crisis mental healthcare for common adult mental health conditions, which are currently delivered by 63 provider NHS trusts.^3^

A separate formula for mental health services was first produced in 1996. The formula has been reviewed on several occasions since,^9,10^ most recently in 2012 when the ‘person-based resource allocation for mental health’ (PRAM)^11^ formula was developed. This applied an approach similar to the person-based resource allocation methodology.^12^ In this paper, we describe how we used linked person-level data for all adults registered with a general practitioner (GP) practice in England to produce a revised formula, which has informed CCG allocations for 2019/20 to 2023/24.^8^ The revised formula is estimated at person level, covers all CCG-commissioned secondary mental health services (including acute in-patient care, community crisis and dementia care, and psychological therapies) and is based on a single prediction model for the whole adult population, in line with the methodology used for the most recent ‘general and acute’ formula.^12,13^

## Method

### Data

We linked routinely available person-level data on use and cost of mental healthcare services in 2015 to need and supply predictors at the individual, area and GP practice level over 2013 and 2014, the two previous years (based on financial years, i.e. 1 April–31 March). Person-level data were extracted and merged via unique NHS numbers by a team in the NHS England Operations and Information Directorate. GP practice- or small-area-level variables were subsequently linked in. After completing the data preparation we retained all registered patients aged 20 years or older, for comparability with PRAM.^11^

Person-level data were extracted from various sources and linked into the list of patients registered with a GP in England and alive at 1 April 2015, which was derived from the Master Person Index (MPI). Information on mental health service use in 2015 was extracted from the Mental Health and Learning Disabilities Data Set (MHLDDS) (intellectual disability is known as learning disability in UK health services), covering 1 April 2014 to 31 December 2015, and the Mental Health Services Data Set (MHSDS), covering 1 January 2016 to 31 March 2016.^14^ Care contacts were classified by the pay band of the care professional who provided them, defined according to the Agenda for Change.^15^ We quantified separately general and intensive in-patient bed-days and we excluded bed-days in low-, medium- and high-security wards, as these are specialised services not commissioned by CCGs.^16^ We used records from the in-patient and out-patient Secondary Uses Service (SUS) data-set^17^ to identify and quantify in-patient stays and care contacts not recorded in the MHSDS or MHLDDS. We extracted and quantified contacts with psychological therapy services from the Improving Access to Psychological Therapies (IAPT) data-set.^18^ Individual demographic and household characteristics (based on age and gender of individuals residing in the same address) were derived from the Master Patient Index. Ethnicity was obtained from SUS, MHSDS, MHLDDS or IAPT data-sets, and physical health diagnoses associated with severe mental illness^19^ were obtained from SUS out-patient data-set records. Additional details on the extraction and linkage of individual-level data are provided in supplementary Appendix 1 available at https://doi.org/10.1192/bjp.2019.185.

Need and supply variables measured in 2013 and 2014 at the level of the GP practice or small geographical area (lower-level super output area, LSOA) were subsequently attributed to individuals according to their GP practice of registration or to their area of residence. We tested ‘attributed variables’ that were either previously used in PRAM^11^ or suggested by NHS England advisory groups for inclusion in our model. We included: the proportion of the population residing in a given LSOA and receiving out-of-work benefit (May 2014–February 2015);^20^ the distance (driving time) from the LSOA centroid to the closest mental health trust headquarters, calculated based on geo-coordinates; a binary indicator for whether the person is registered with a student GP practice (with a proportion of young people higher than 40% or located in proximity of university or colleges sites); and the prevalence of severe mental illness for GP practice, as recorded in the GP Quality and Outcomes Framework (QOF) in 2014.

We generated a set of binary indicators for whether the individual was registered at 1 April 2015 with a GP practice in each of the 211 CCGs existing at the time. We also generated a set of variables indicating the share of patients registered with a given GP practice who had been in contact at least once with each of the 66 NHS trusts providing mental healthcare over the financial years 2013 or 2014.

### Costing

For each individual we calculated the total mental healthcare cost in 2015. This was the sum of the costs associated with in-patient bed-days, community care contacts and IAPT contacts.

We retrieved from the national schedule of NHS Reference Costs 2015/16^21^ the unit cost and the volume of admitted and non-admitted (non-secure and non-specialised) mental healthcare days and IAPT episodes per cluster and we multiplied them to derive the relative total cost. A cluster is a grouping of patients with the same level of risk and therefore clinical and resource need.^22^ Although the costs for mental health and IAPT initial assessments was provided in the Reference Costs, we did not include them as we could not distinguish these accurately in the MHSDS and MHLDDS. We multiplied the unit cost and the volume to derive the cost per cluster of admitted and non-admitted care days and we matched it with the volume of bed-days and community care contacts recorded in the MHLDDS between 1 April and 31 December 2015 to obtain their unit costs. We assumed constant service delivery over the year and we multiplied the annual cost of admitted and non-admitted care days per cluster by 0.75, to obtain the equivalent for 1 April and 31 December 2015.

We calculated the average unit costs for general and for intensive bed-days by matching the total costs with data from the MHLDDS on patients' cluster assignment and length and clinical intensity of all ward-stay episodes. We weighted intensive bed-days as 1.97 general bed-days, based on the ratio of intense to general bed-day unit costs reported in the NHS Reference Costs 2011/12,^23^ which reported bed-day cost by intensity. We divided the cost of admitted care days per cluster by the weighted sum of general and intensive bed-days to obtain the cluster-specific unit cost of a general in-patient bed-day, which we multiplied by 1.97 to obtain the cluster-specific unit cost of an intensive bed-day. We averaged across clusters, weighting for the number of bed-days within each cluster, to obtain the unit cost of general (£371) and intensive (£752) bed-days.

We calculated the average unit cost of care contacts by matching the total cost with data from the MHLDDS on each patient's cluster assignment and the care professional job role, occupation code and specialty for each care contact. We mapped the job role, occupation code and specialty of the healthcare professional to a pay band according to the latest NHS Agenda for Change pay scale.^15^ We weighted each contact by the ratio of the mid-point salary of the pay band to the mid-point salary of band two. Information on the professional providing care was missing for 2.7% of the care contacts. To validate the pay band attribution, we used information on the salary of the healthcare professional from the currency development project for children and young people's mental health. The project is currently carried out by NHS England using data from nine pilot mental health trusts to test and develop grouping of children and young people seeking mental health support and with broadly similar resource needs.^24^ We also used this information to attribute an average salary to professionals with missing information on job role, occupation code and specialty (pay band six). We divided the total cost of non-admitted care days by the weighted sum of contacts within each cluster and we multiplied by the contact weight to obtain the average unit cost of a care contact by cluster. We averaged across clusters, weighting for the number of contacts within each cluster to derive the average unit costs of contacts with care professionals in pay band two (£109), three (£117), four (£134), five (£158), six (£194), seven (£230), eight (£274) or nine (£274).

To calculate the average unit cost of an IAPT consultation (£94) we divided the total cost across clusters by the number of low- and high-intensity contacts, excluding initial assessment, as reported in the NHS Reference Costs 2015/16.^21^

### Estimating predictors of costs

We estimated individual mental healthcare costs in 2015 as a function of need and supply variables in 2013 and 2014 using a linear regression model (ordinary least squares) with robust standard errors.^13^ We truncated the total cost at £100 000 for any individual, to avoid bias in the estimated coefficients due to outliers.^11^ As individual-need indicators, we included the interactions between gender and 5-year age bands, and sets of binary indicators for ethnicity, for household type and for physical health diagnoses. We included as attributed-need variables the proportion of the LSOA population receiving out-of-work benefit, as an indicator of worklessness and deprivation, registration with a student GP practice and GP practice severe mental illness prevalence. We included the distance from the closest mental health trust headquarters, a set of indicators for GP practice share of patients in contact with each mental health trust, and a set of CCG binary indicators to control for differences in supply. Distance was calculated as driving time based on Ordnance Survey road data, with average road speeds by road type using Routefinder for MapInfo.

We estimated the mental healthcare cost for individuals registered with a random 50% of GP practices and we used the remaining 50% as a validation sample. We predicted the cost for each individual and we aggregated to get the total for each GP practice within both samples. We compared the model with alternative ones using three measures of predictive performance calculated separately on the estimation and on the validation sample: the coefficient of variation (*R*^2^), the mean absolute prediction error and the proportion of GP practice predictions not within 10% of the actual cost.^13^ The first model was estimated including the set of variables most similar to those used in PRAM, removing variables without a significant effect on mental healthcare cost or GP practice- and LSOA-level variables that could be replaced by individual-level variables. Additional variables were added in the model on the basis of the literature and on the advice of clinical and epidemiological experts. They were retained if the estimated coefficient was significant and, for attributed variables, of the expected sign. We avoided the inclusion of variables that could generate perverse incentives, for example, to over-record diagnoses. For simplicity and to avoid overfitting, more parsimonious models were preferred among those with similar statistical robustness and predictive power. Variables that would appear to explain most variation, with more precisely estimated coefficients, were retained.

### Calculating CCG need indices

We produced individual-need weights by taking predictions from the person-level model with the supply variables fixed at their population average values. This sterilises the effect of variations in access to care on the need-based target allocations. Any variation in prediction would therefore reflect differences in need variables. We similarly sterilised the effect of variables whose estimated coefficients indicated unmet need, namely ethnic groups. Without clinical explanation provided in the literature or by experts consulted in NHS England, the negative coefficient could indicate unmet need in the use of mental healthcare.^25^ The inclusion of these variables does not affect the allocations directly but has indirect effects by changing the coefficients on other variables with which they are correlated.^25^

We compared the distribution of need with the distribution of actual costs across GP practices using three measures calculated on the whole population, including estimation and validation samples.^13^ The redistribution index is the proportion of the total budget redistributed from ‘losing’ practices to ‘gaining’ practices and takes a value of 0–0.5. The redistribution index is half the sum across all GP practices of the absolute differences between the shares of need and the shares of actual cost. The mean absolute percentage change in share is the average across GP practices of the absolute difference between the share of actual cost and the share of need, divided by the shares of actual cost. The proportion of practice shares substantially affected indicates the proportion of GP practices whose absolute percentage change in share is at least 5%.

We generated need weights for the capitation formula by averaging the individual-need estimates by GP practice, age and gender strata, which we multiplied by the number of registered patients in each stratum (average between November 2017 and October 2018). These were summed to create GP practice raw weighted populations, which we then normalised to the total population registered with a GP in England to create GP practice normalised weighted populations, then aggregated to CCG level. A CCG's need index is its weighted population divided by its unweighted population and provides an indication of need relative to other CCGs. We plotted the CCG need weights against the Indices of Multiple Deprivation 2015^26^ to see whether more deprived areas received a higher weight. The analysis was carried out using Stata 14 for Windows.

### Ethics statement

The study uses routinely collected anonymised administrative data and did not affect the type of care that patients received. Consent by patients using the service was not required.

## Results

Out of the 43 751 535 registered patients aged 20 years or older, 4.01% had some contact with secondary mental health and/or IAPT services in 2015. The average cost per registered patient was £80.60. The cost per patient in contact with mental health/IAPT services ranged from £94 to £1 040 963 and was £2008.46 on average. The average cost per registered patient varied between £1.66 and £1847 across GP practices, except for one GP practice where it was 0. Table A1 in supplementary Appendix 2 provides summary statistics of individual- and area-level characteristics for the whole population and only for those in contact with mental health services.

Table 1 presents the coefficients associated with each need and supply variable included in the analysis. The coefficients associated with the age and gender groups indicate that mental healthcare costs increased between 20 and 45 years of age and then decreased steadily until 75 years and more sharply after 85 years. The cost was higher for men than women between 20 and 30 years of age and *vice versa* between 30 and 65 years of age.

**Table 1:** Effect of need and supply variables on mental healthcare cost

	Coefficient	95% CI	*P*-value
Age band, gender (base category: 20–24 years, female)			
20–24 years, male	11.811	7.371 to 16.251	0.0000
25–29 years, female	−0.455	−4.241 to 3.332	0.8140
25–29 years, male	12.574	8.083 to 17.065	0.0000
30–34 years, female	8.312	4.493 to 12.130	0.0000
30–34 years, male	13.196	8.817 to 17.575	0.0000
35–39 years, female	17.607	13.720 to 21.495	0.0000
35–39 years, male	14.089	9.668 to 18.510	0.0000
40–44 years, female	22.547	18.626 to 26.469	0.0000
40–44 years, male	14.297	10.107 to 18.488	0.0000
45–49 years, female	13.809	10.025 to 17.594	0.0000
45–49 years, male	2.851	−1.041 to 6.742	0.1511
50–54 years, female	3.515	−0.309 to 7.339	0.0716
50–54 years, male	−8.151	−12.018 to −4.285	0.0000
55–59 years, female	−11.087	−15.062 to −7.111	0.0000
55–59 years, male	−19.567	−23.629 to −15.505	0.0000
60–64 years, female	−29.366	−33.294 to −25.438	0.0000
60–64 years, male	−28.665	−32.936 to −24.394	0.0000
65–69 years, female	−26.666	−30.989 to −22.342	0.0000
65–69 years, male	−29.11	−33.582 to −24.638	0.0000
70–74 years, female	−22.038	−27.119 to −16.956	0.0000
70–74 years, male	−24.743	−29.964 to −19.522	0.0000
75–79 years, female	−22.513	−28.349 to −16.678	0.0000
75–79 years, male	−19.835	−25.620 to −14.051	0.0000
80–84 years, female	−28.236	−34.822 to −21.649	0.0000
80–84 years, male	−5.964	−13.725 to 1.797	0.1320
85 years or older, female	−91.622	−97.581 to −85.663	0.0000
85 years or older, male	−51.257	−58.320 to −44.195	0.0000
Ethnicity (base category: White British)			
Irish	33.914	20.819 to 47.009	0.0000
Any other White background	−24.562	−28.243 to −20.881	0.0000
White and Black Caribbean	140.276	108.048 to 172.505	0.0000
White and Black African	53.718	18.928 to 88.508	0.0025
White and Asian	79.916	44.033 to 115.798	0.0000
Any other mixed background	17.851	2.698 to 33.003	0.0209
Indian	−21.811	−27.336 to −16.287	0.0000
Pakistani	−15.173	−22.627 to −7.720	0.0001
Bangladeshi	−23.162	−35.369 to −10.955	0.0002
Any other Asian background	−6.963	−15.034 to 1.108	0.0909
Caribbean	133.693	118.540 to 148.846	0.0000
African	29.735	19.425 to 40.045	0.0000
Any other Black background	124.854	108.283 to 141.425	0.0000
Chinese	−49.287	−58.808 to −39.766	0.0000
Any other ethnic group	−18.62	−24.810 to −12.429	0.0000
Household type (base category: two adults of opposite gender)			
Care home	436.937	410.665 to 463.209	0.0000
Missing	59.225	54.736 to 63.715	0.0000
Multi-adult	−9.11	−10.727 to −7.493	0.0000
Multi-adult and one or more children	−39.761	−41.663 to −37.859	0.0000
Multi-child	−37.547	−61.615 to −13.480	0.0022
Other communal	146.814	131.932 to 161.695	0.0000
One adult and one or more children	−21.878	−25.034 to −18.722	0.0000
Single person	101.47	98.753 to 104.187	0.0000
Two adults and one or more children	−42.885	−44.765 to −41.006	0.0000
Two adults of the same gender	29.895	25.949 to 33.840	0.0000
Physical health			
Viral hepatitis (ICD-10 codes B15–B19)	284.688	204.185 to 365.192	0.0000
Symptoms and signs involving cognition, perception, emotional state and behaviour (ICD-10 codes R40–R46)	838.115	800.583 to 875.647	0.0000
Poisoning by, adverse effect of and underdosing of drugs, medicaments and biological substances (ICD-10 codes T36–T50)	1698.611	1623.271 to 1773.951	0.0000
Diabetes mellitus (ICD-10 codes E10–E14)	70.49	62.606 to 78.375	0.0000
Endocrine, nutritional and metabolic diseases (ICD-10 codes E15–E90)	63.484	57.044 to 69.924	0.0000
Cerebrovascular diseases (ICD-10 codes I60–I69)	52.102	27.494 to 76.710	0.0000
Chronic lower respiratory diseases (ICD-10 codes J40–J47)	72.956	67.041 to 78.870	0.0000
Attributed need variables			
Proportion receiving out-of-work benefits in LSOA	269.048	253.966 to 284.130	0.0000
Severe mental illness prevalence in GP practice	22.323	18.448 to 26.198	0.0000
Student GP practice	−28.164	−34.305 to −22.024	0.0000
Attributed supply variables			
LSOA drive time from closest MH trust^a^	−0.331	−0.405 to −0.257	0.0000
CCG indicators^a^	Yes		
Indicators of usage of each provider by GP practices^a^	Yes		
Constant	Yes		
Adjusted *R*^2^	0.008		
Observations	21 319 709		

LSOA, lower-level super output area; CCG, clinical commissioning group; GP, general practitioner.

a. Denotes supply variables. Coefficients are not standardised, so they are dependent on the units of measurement.

Compared with White British, other ethnic groups had higher average costs per year, by at least £34 (Irish). Costs were substantially higher for the White and Black Caribbean (£140 higher), Black Caribbean (£134 higher) and ‘any other Black background’ (£125 higher) groups, suggesting higher need for care among these groups.

Compared with individuals living in a two-adult opposite-gender household, individuals living alone had higher cost (£101), as did individuals living in communal households (£147) or in care homes (£437). Individuals living in households with two or more adults and/or children had lower cost.

The cost was higher for individuals who had experienced at least one admission with a diagnosis of poisoning by drugs, medicaments or biological substances (£1699) or of viral hepatitis (£285). The cost was also moderately higher for individuals who had an admission with a diagnosis of diabetes (£70), endocrine, nutritional or metabolic disease (£63), cerebrovascular disease (£52) or chronic lower respiratory disease (£73). Previous admissions with symptoms and signs involving cognition, perception, emotional state and behaviour were also associated with higher mental health costs (£838).

Residing in an LSOA with a higher proportion of individuals receiving out-of-work benefit was associated with increased cost (£2.70 per extra percentage point), as was being registered with a GP practice with higher prevalence of severe mental illness (£22 per extra percentage point). Being registered with a student GP practice was associated with a £28 lower cost, indicating lower need for mental healthcare compared with a population of similar age and socioeconomic conditions but registered with a practice with a different patient list composition.

An extra 10 minutes' driving time to the headquarters of the closest mental health trust reduced the cost by £3.30, indicating that poorer access was associated with lower costs of mental healthcare.

The model explained a high proportion of the variation in mental healthcare costs at the GP practice level in both the estimation (*R*^2^ = 0.81) and the validation samples (*R*^2^ = 0.79), and at the CCG level (*R*^2^ >0.99). As illustrated in Table 2, the predictive power of the selected model on the validation sample, evaluated at the GP practice level, was higher compared with the alternative models. These models included age and gender only (*R*^2^ = 0.40), all need variables at the individual level (*R*^2^ = 0.48), all need variables at the individual, LSOA and GP practice level (*R*^2^ = 0.54), and all need and supply variables (*R*^2^ = 0.54). The inclusion of the CCG indicators and the GP practice share of patients in contact with each mental health trust explained an additional 25% of the variation. Distance from the closest mental health trust was included to improve the precision of the estimated coefficients and of the need predictions but did not increase the model's predictive power. In a model containing need and supply variables, the need variables alone predicted 52% of the variation in cost across GP practices.

**Table 2:** Model predictive and redistributive performance

	Age and gender only	Individual-level need variables	Individual- and area-level need variables	Individual- and area-level need and supply variables	All variables
Predictive performance					
*R*^2^ for estimation sample	0.3886	0.454	0.5117	0.5162	0.8078
Mean absolute error for estimation sample	200 000	180 000	170 000	170 000	100 000
*R*^2^ for validation sample	0.3986	0.476	0.5366	0.542	0.7929
Mean absolute error for validation sample	180 000	170 000	160 000	160 000	100 000
Proportion of GP practice predictions not within 10%	0.8633	0.8513	0.8352	0.8331	0.8297
Redistributive performance					
Redistribution index	0.2201	0.2017	0.1875	0.1872	0.1879
Mean absolute percentage change in share	74.181	68.4718	62.6231	62.6433	63.7975
Proportions of GP practice shares substantially affected	0.9283	0.9234	0.9152	0.9147	0.918

GP, general practitioner.

The all-variables redistribution index (0.1896) indicated that 19% of the total budget should be redistributed for the distribution of mental healthcare cost in 2015 to match the distribution of need across GP practices. For 92% of the GP practices the absolute difference in need and cost shares would be higher than 5%, as indicated by the proportions of GP practice shares substantially affected (0.918). When calculated across CCGs, the redistribution index indicated that 14% of the total budget should be redistributed to match the distribution of need.

**Fig. 1:**
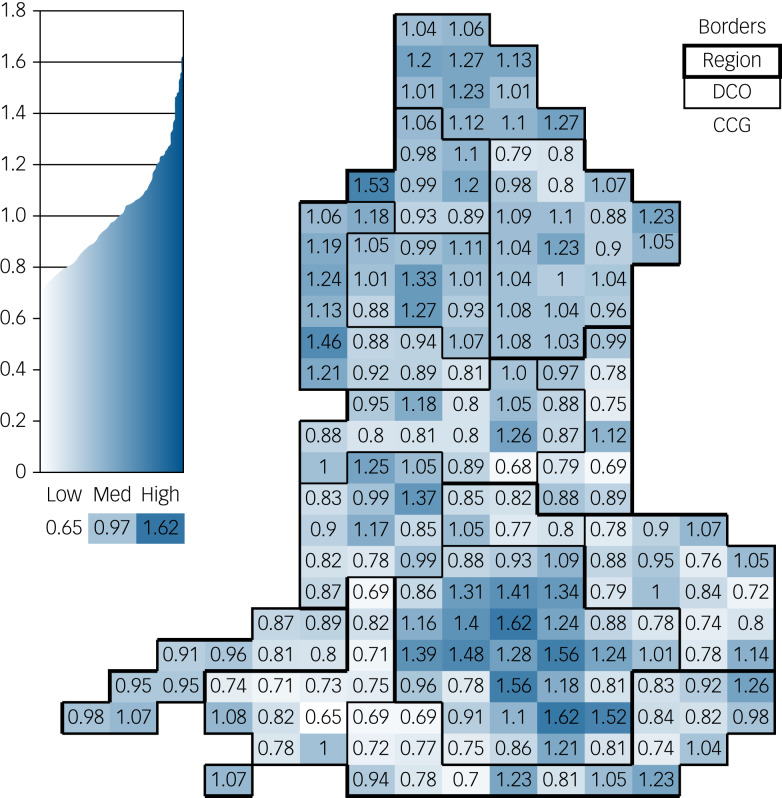
Clinical commissioning group (CCG) need indices for 2018, as derived from the revised model.

The CCG need indices derived from the model highlight the geographic variability in need across England (Fig. 1). The need indices show the relative weight given to patients across CCGs. They vary around 1.00, reflecting relative differences in the underlying need captured by the variables included in the model. The CCG with the lowest relative need for mental health services has only 65% of the average need per person in England. The highest-need CCG has average needs that are 62% higher than the national average. These need indices, when combined with those produced separately for general and acute, maternity and community mental health services, and for prescribing and unmet need, inform a CCG's target share of the overall budget.^6–8^

DCO, Director of Commissioning Operations – Local Office.

**Fig. 2:**
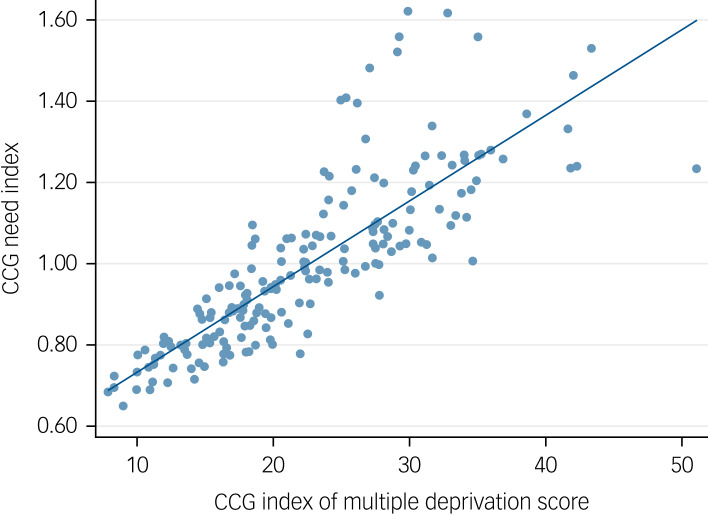
Clinical commissioning group (CCG) need index by CCG Index of Multiple Deprivation score in 2015.^26^

Need indices are higher in more deprived CCGs, as illustrated in Fig. 2. The highest need indices are in urban centres with younger, deprived populations. The need indices for each of the 192 CCGs in existence in January 2019 are available in supplementary Appendix 3.

## Discussion

We used linked person-level data for all adults registered with a GP practice in England to model an updated formula for secondary mental healthcare, learning disability (intellectual disability) and IAPT services. We applied a person-based methodology fully aligned with the one used for other components of CCG allocations to generate weights for the capitation formula. The developed model included individual-, area- and GP practice-level need variables and supply variables, namely distance from the closest mental health trust, the CCG where the patient is registered and the GP practice share of patients in contact with mental health trusts. The model explains up to 81% of the variation in cost across GP practices, with need variables alone explaining 52% of the variation. It explains over 99% of the variation in cost at CCG level. The use of linked person-based data allowed us to identify person-level predictors of costs that could be used in the formula instead of corresponding area-level variables.

### Strengths of the revised model

Compared with the PRAM model, the formula developed is estimated on more accurate data and based on a simpler model with higher predictive power. It is therefore more likely to identify relative need correctly and produce equitable allocations. First, it is more comprehensive and includes learning disability and IAPT services. Second, it is based on fully linked person-level data for all patients registered with a GP practice, rather than only for patients using mental healthcare services. Although the PRAM model estimated the probability of using services at the group level, we were able to estimate a single model for mental healthcare costs for the whole population. We could also include ethnic background and household type at individual level rather than at area level. The use of person-level information led to the exclusion of area- and GP practice-level need variables used in previous models, as they no longer contribute additionally to the explained variation.

### Variables omitted from the revised model

The PRAM model included diagnoses of mental health disorders and risk flags derived from the MHSDS or MHLDDS, namely a previous in-patient stay of at least two nights and previous treatment from a number of different types of health professional. Although the inclusion of diagnoses and risk indicators increased the predictive power of the model,^8,9^ we did not include them because of substantial heterogeneity in data reporting across providers. This would have led us to underestimate need in areas served by providers who reported information incompletely. Moreover, with relatively weak controls on data quality, their inclusion could generate incentives to over-record diagnoses in future. We tested the inclusion of three variables derived from the SUS Accident and Emergency Data Set indicating whether the person had attended an accident and emergency department with a diagnosis of mental health disorder. Despite the large and significant coefficients, suggesting a strong association with mental healthcare cost, we also did not include these variables, because of the high variability in reporting across trusts.

### Areas for improvement of the model

The development of the model was limited by the data quality in several aspects, which points to areas for future improvement. First, the unit cost was not differentiated by the assignment of patients to different clusters, which correspond to different care pathways and cost. As data quality improves and becomes more homogeneous, costed service categories could be refined by cluster assignment and the exclusion of specialised services could be refined. Second, diagnoses of mental health disorders, mental health risk-indicators and accident and emergency diagnoses could be reintroduced in the model. Third, appropriate methods for dealing with heterogeneity in reporting of diagnoses could be developed, for example by relying on diagnoses notoriously more consistently reported or by identifying and computing adjustments for individuals served by under-reporting providers.

Disentangling the effects of need and supply influences on healthcare use is crucial to improving the capacity of utilisation-based models to estimate need.^27^ Although we have conditioned on the percentage of each GP practice's patients in contact with each provider to capture differences in access, additional variables capturing differences in supply and access to care across and within areas should be identified and included in future models. Need indicators such as person-level primary care diagnoses, employment and housing status and income and wealth could also be included, should linkage with primary care and non-health data-sets at the person level become feasible.

The model does not cover children and young people, but as data become available in the MHSDS, a person-based model, and appropriate strategies to address potential unmet need, could be developed for this population.

### Current use of the revised model

The refinement of the formula used to allocate resources for mental healthcare in England is a continual process. The need indices produced from these models give a high weight to urban centres with deprived populations. Compared with the PRAM model, this model attributes a higher weight to areas with more deprived and older populations. These models have informed the distribution of some 12% (£9 bn in 2019/20) of the health budget allocated to CCGs over the next 5 financial years (2019/20 to 2023/24).

## Supplementary material

For supplementary material accompanying this paper visit https://doi.org/10.1192/bjp.2019.185. click here to view supplementary material
