# Supplementary material for: Estimating local need for mental healthcare to inform fair resource allocation in the NHS in England: cross-sectional analysis of national administrative data linked at person level
Source: Br J Psychiatry. 2020 Jun;216(6):338–44. doi: 10.1192/bjp.2019.185 (PMC7511896; doi:10.1192/bjp.2019.185)
Supplement: Supplementary file 1 [file S0007125019001855sup001.docx]

**Appendix 1: Individual level data extraction and linkage**

We linked routinely available person level data on use and cost of mental health care services in 2015, to need and supply predictors at the individual, area and GP practice level over 2013 and 2014, the two previous years. We referred to financial years (1 April to 31 March). Person level data were extracted from different datasets, as illustrated in this appendix, and merged via unique NHS numbers by a team in the NHS England Operations & Information Directorate.

*Master Person Index*

The Master Person Index (MPI) has been developed within NHS England’s data services and includes person level information from the National Health Applications and Infrastructure Services (NHAIS) for all patients registered, or who have been registered, with a GP in England and Wales [[1](#_ENREF_1)]. We retained only individuals registered with a GP practice in England and alive at 1 April 2015, therefore excluding all patients not active or who moved out of England. We retained information at 1 April 2015 on age (derived from the month of birth), gender, GP practice of registration and LSOA of residence. From the MPI we derived a variable, based on the postcode and the age and gender of individuals co-residing, which indicates the composition of the household that the individuals live in. We distinguished between: care home or other communal establishment; multi-adult; multi-adult and one or more children; two adults of the same gender; two adults of different gender; two adults and one or more children; one adult and one or more children; or single person. The 'care home' household type is based on residence in a care home categorised as such by the Care Quality Commission (CQC), NHS Digital or other sources. Multi-adult households may capture a mix of young adult flat-shares along with those sharing for other reasons (such as supported living).

*Mental health dataset*

We derived information on the use of mental health services from two datasets: the Mental Health and Learning Disabilities Dataset (MHLDDS) covering 1 April 2014 to 31 December 2015 and the Mental Health Services Dataset (MHSDS) covering 1 January 2016 to 31 March 2016. MHLDDS and MHSDS cover individuals in contact with secondary mental health and learning disability services, provided in hospitals, outpatient clinics and community [[2](#_ENREF_2)]. Learning disabilities were not excluded as patients with learning disabilities received at least once care for mental health and episodes of care and contacts with care professionals due to learning disabilities could not be identified in a consistent way. Episodes of care could be identified through clinical intensity of inpatient care, treatment function or specialty, while contacts of care through the job role or the specialty of the care professional, or through the main reason for referral. There would have been discrepancies in the way that different providers complete these fields in the MHSDS and MHLDDS. Moreover, information on the main reason for referral was only available in the more recent data and poorly completed.

We used data from the MHSDS for the period between 1 January and 31 March 2016 only to quantify individual inpatient and community service use. Due to disruptions during the transition from MHLLDS to MHSDS, there was a drop in the quality of data reporting. While we could verify the reporting of the volume of service provided through Secondary Uses Service data, we could not verify the quality of other variables, which we avoided using.

The total number of inpatient bed days per person was capped at 365. The total is calculated as the sum of bed days within each reported ward stay, which is based on the reported start and end date of each ward stay, or, when these were missing, on the beginning or end of the financial year. We differentiated between intensive (intended clinical intensity codes 11, 51, 61) and general (non-intensive intended clinical intensity codes 12, 52, 53, 62 and 63 or unknown intended clinical intensity code) care [[3](#_ENREF_3)]. We excluded bed days in low, medium and high security level wards as these are specialised services not commissioned by CCGs [[4](#_ENREF_4)]. We counted contacts with different types of health care professionals, including consultations face-to-face, by telephone, by web-cam or needing a translator and that were attended on time or attended late but the patient could be seen. Health care professionals were classified by pay band (two to nine) based, in order of priority, on their job role, occupation code or specialty^[[1]](#footnote-1)^. Each job role, occupation code and specialty was attributed a pay band according to Agenda for Change [[5](#_ENREF_5)], NHS job adverts, and the judgement of experienced NHS colleagues.

*Improving Access to Psychological Therapies (IAPT) dataset*

The Improving Access to Psychological Therapies (IAPT) Data Set collects information about adults in contact with psychological therapy services. We counted the number of consultations face–to-face, by telephone, by webcam, or needing a translator, and that were attended or attended late, but the patient would be seen [[6](#_ENREF_6)].

*Secondary Uses Service dataset*

The Secondary Uses Service (SUS) is the single, comprehensive repository for healthcare data in England. We extracted information on physical health diagnoses from records of patients admitted to general and acute care hospitals [[7](#_ENREF_7)]. We generated seven flags that indicate if the person had been admitted in 2013 or 2014 at least once with each of the following diagnoses defined based on ICD-10 codes: viral hepatitis (B15-B19); symptoms and signs involving cognition, perception, emotional state and behaviour (R40-R46); poisoning by adverse effect of and under dosing of drugs, medicaments and biological substances (T36-T50); diabetes mellitus (E10-E14); endocrine nutritional and metabolic diseases (E15-E90); cerebrovascular diseases (I60-I69); and chronic lower respiratory diseases (J40-J47). We considered the primary or secondary diagnoses up to the 7^th^ position in total, as in the formula for General and Acute care [[8](#_ENREF_8)]. The selected diagnoses were either used in the PRAM review or known to be associated with severe mental illness [[9](#_ENREF_9)].

We used SUS data to verify and refine the mental health care service use reported in the MHLDDS and MHSDS. For each individual we extracted inpatient admissions and outpatient consultations from SUS admitted and outpatient care records related to mental health. We identified the spells of care and the consultations related to mental health (and their records) through the spell core Health Related Group (PA52Z, PA52B, PA52C, PA53A, PA53B, VC26Z, VC28Z), the treatment function or the main specialty code (700, 710, 711, 712, 713, 715, 720, 721, 722, 723, 724, 725, 726, 727). We checked the overlap between inpatient stays and between outpatient consultations as reported in SUS and in the MHSDS (1 January 2016 to 31 March 2016) or MHLDDS (1 April 2014 to 31 December 2015). We identified overlap by patient and by dates of inpatient stay admission and discharge, or by date of outpatient consultations. We added the non-overlapping spells to the count of general inpatient bed days and the count of non-overlapping outpatient consultations to the count of contacts with a health care professional in pay-band 7.

*Ethnicity*

Ethnicity was derived in order of priority from SUS inpatient, SUS outpatient, SUS Accident and Emergency, MHSDS, MHLDDS and IAPT records, for 70% of individuals in the MPI who used services and had a corresponding record between 1 April 2013 and 31 March 2016. We merged ethnicity with the MPI records and we derived a set of binary indicators for ethnic group categories. A value equal to the LSOA proportion of residents in each ethnic category was imputed for the 30% of individuals with missing information on ethnicity.

**References**

1. NHS Digital. *National Health Applications and Infrastructure Services*. 2018; Available from: https://digital.nhs.uk/services/systems-and-service-delivery/national-health-application-and-infrastructure-services/national-health-application-and-infrastructure-services-nhais-more-services.

2. NHS Digital. *Mental Health Services Datataset*. 2018; Available from: https://digital.nhs.uk/data-and-information/data-collections-and-data-sets/data-sets/mental-health-services-data-set.

3. NHS Digital. *NHS Data Dictionary, Intended Clinical Intensity Code*. 2018; Available from: <http://www.datadictionary.nhs.uk/data_dictionary/data_field_notes/i/in/intended_clinical_care_intensity_code_(mental_health)_de.asp?query=intended%20intensity%20code&rank=1&shownav=1> ; <http://www.datadictionary.nhs.uk/data_dictionary/attributes/c/cla/clinical_care_intensity_de.asp?shownav=1>.

4. NHS England, *Manual for Prescribed Specialised Services 2017/18*, 2017, NHS England Analytical Services (Finance): London.

5. NHS Employers, *Agenda for Change pay scales - Annual*, 2017, NHS Employers: London.

6. NHS Digital. *Improving Access to Psychological Therapies Data Set*. 2018; Available from: https://digital.nhs.uk/data-and-information/data-collections-and-data-sets/data-sets/improving-access-to-psychological-therapies-data-set.

7. NHS Digital. *Secondary Uses Service (SUS)*. 2018; Available from: https://digital.nhs.uk/services/secondary-uses-service-sus.

8. Chaplin, M., et al. *Refreshing the Formulae for CCG Allocations For allocations to Clinical Commissioning Groups from 2016-17, Report on the methods and modelling*. 2015; Available from: https://[www.england.nhs.uk/wp-content/uploads/2016/04/3-rep-elland-all-sections.pdf](http://www.england.nhs.uk/wp-content/uploads/2016/04/3-rep-elland-all-sections.pdf)

9. Public Health England, *Severe mental illness (SMI) and physical health inequalities: briefing*, 2018, Public Health England: London, 27 September 2018.

**Appendix 2: Summary statistics**

**Table A1 - Mental health service use and cost (2015) and need and supply variables (2013-2014)**

|  | **All adults** | | | **Users only** | | |
| --- | --- | --- | --- | --- | --- | --- |
|  | **Mean** | **Min** | **Max** | **Mean** | **Min** | **Max** |
| Mental Health care user | 0.040 | 0 | 1 |  |  |  |
| Cost (£) per person | 80.598 | 0 | 1,040,963 | 2,008.46 | 94 | 1,040,963 |
|  |  |  |  |  |  |  |
| Male | 0.493 | 0 | 1 | 0.406 | 0 | 1 |
| Age |  |  |  |  |  |  |
| 20-24 years | 0.082 | 0 | 1 | 0.101 | 0 | 1 |
| 25-29 years | 0.091 | 0 | 1 | 0.104 | 0 | 1 |
| 30-34 years | 0.093 | 0 | 1 | 0.099 | 0 | 1 |
| 35-39 years | 0.086 | 0 | 1 | 0.089 | 0 | 1 |
| 40-44 years | 0.089 | 0 | 1 | 0.093 | 0 | 1 |
| 45-49 years | 0.094 | 0 | 1 | 0.091 | 0 | 1 |
| 50-54 years | 0.090 | 0 | 1 | 0.084 | 0 | 1 |
| 55-59 years | 0.077 | 0 | 1 | 0.063 | 0 | 1 |
| 60-64 years | 0.068 | 0 | 1 | 0.045 | 0 | 1 |
| 65-69 years | 0.070 | 0 | 1 | 0.041 | 0 | 1 |
| 70-74 years | 0.053 | 0 | 1 | 0.038 | 0 | 1 |
| 75-79 years | 0.042 | 0 | 1 | 0.045 | 0 | 1 |
| 80-84 years | 0.032 | 0 | 1 | 0.048 | 0 | 1 |
| 85 years or older | 0.035 | 0 | 1 | 0.061 | 0 | 1 |
| Ethnicity |  |  |  |  |  |  |
| White British | 0.719 | 0 | 1 | 0.801 | 0 | 1 |
| Irish | 0.006 | 0 | 1 | 0.008 | 0 | 1 |
| Any other White background | 0.052 | 0 | 1 | 0.043 | 0 | 1 |
| White and Black Caribbean | 0.003 | 0 | 1 | 0.004 | 0 | 1 |
| White and Black African | 0.001 | 0 | 1 | 0.001 | 0 | 1 |
| White and Asian | 0.002 | 0 | 1 | 0.002 | 0 | 1 |
| Any other mixed background | 0.005 | 0 | 1 | 0.005 | 0 | 1 |
| Indian | 0.022 | 0 | 1 | 0.016 | 0 | 1 |
| Pakistani | 0.017 | 0 | 1 | 0.014 | 0 | 1 |
| Bangladeshi | 0.006 | 0 | 1 | 0.005 | 0 | 1 |
| Any other Asian background | 0.014 | 0 | 1 | 0.011 | 0 | 1 |
| Caribbean | 0.010 | 0 | 1 | 0.014 | 0 | 1 |
| African | 0.014 | 0 | 1 | 0.011 | 0 | 1 |
| Any other Black background | 0.007 | 0 | 1 | 0.009 | 0 | 1 |
| Chinese | 0.004 | 0 | 1 | 0.002 | 0 | 1 |
| Any other ethnic group | 0.022 | 0 | 1 | 0.020 | 0 | 1 |

**Table A1 –Mental health service use and cost (2015) and need and supply variables (2013-2014)**  **(continued)**

|  | **All adults** | | | **Users only** | | |
| --- | --- | --- | --- | --- | --- | --- |
|  | **Mean** | **Min** | **Max** | **Mean** | **Min** | **Max** |
| Physical health diagnoses |  |  |  |  |  |  |
| Viral hepatitis (ICD-10 codes B15-B19) | 0.001 | 0 | 1 | 0.002 | 0 | 1 |
| Symptoms and signs involving cognition, perception, emotional state and behaviour (ICD-10 codes R40-R46) | 0.005 | 0 | 1 | 0.027 | 0 | 1 |
| Poisoning by drugs, medicaments and biological substances (ICD-10 codes T36-T50) | 0.002 | 0 | 1 | 0.018 | 0 | 1 |
| Diabetes mellitus (ICD-10 codes E10-E14) | 0.020 | 0 | 1 | 0.037 | 0 | 1 |
| Endocrine nutritional and metabolic diseases (ICD-10 codes E15-E90) | 0.029 | 0 | 1 | 0.053 | 0 | 1 |
| Cerebrovascular diseases (ICD-10 codes I60-I69) | 0.003 | 0 | 1 | 0.009 | 0 | 1 |
| Chronic lower respiratory diseases (ICD-10 codes J40-J47) | 0.030 | 0 | 1 | 0.061 | 0 | 1 |
| Household type |  |  |  |  |  |  |
| Care home | 0.005 | 0 | 1 | 0.027 | 0 | 1 |
| Missing | 0.061 | 0 | 1 | 0.050 | 0 | 1 |
| Multi-adult | 0.254 | 0 | 1 | 0.218 | 0 | 1 |
| Multi-adult and one or more children | 0.123 | 0 | 1 | 0.095 | 0 | 1 |
| Multi-child | 0.000 | 0 | 1 | 0.000 | 0 | 1 |
| Other communal | 0.014 | 0 | 1 | 0.014 | 0 | 1 |
| One adults and one or more children | 0.024 | 0 | 1 | 0.041 | 0 | 1 |
| Single person | 0.132 | 0 | 1 | 0.212 | 0 | 1 |
| Two adults and one or more children | 0.130 | 0 | 1 | 0.114 | 0 | 1 |
| Two adults of different gender (base category) | 0.223 | 0 | 1 | 0.187 | 0 | 1 |
| Two adults of the same gender | 0.033 | 0 | 1 | 0.044 | 0 | 1 |
|  |  |  |  |  |  |  |
| Attributed variables |  |  |  |  |  |  |
| Need |  |  |  |  |  |  |
| Proportion in LSOA receiving out of work benefits | 0.088 | 0.001 | 0.490 | 0.101 | 0.001 | 0.490 |
| Registered with Student GP practice | 0.018 | 0 | 1 | 0.016 | 0 | 1 |
| Prevalence (%) of Severe Mental Illness in GP practice | 0.881 | 0.059 | 15.567 | 0.927 | 0.059 | 15.567 |
| Supply |  |  |  |  |  |  |
| Drive time to closest mental health trust (mins) | 22.606 | 0.28 | 105.83 | 22.084 | 0.280 | 105.83 |

**Appendix 3: CCG Need Indices**

| **CCG code** | **CCG Name** | **Need Index** |
| --- | --- | --- |
| 10C | NHS Surrey Heath CCG | 0.65 |
| 04N | NHS Rushcliffe CCG | 0.68 |
| 99H | NHS Surrey Downs CCG | 0.69 |
| 09Y | NHS North West Surrey CCG | 0.69 |
| 14Y | NHS Buckinghamshire CCG | 0.69 |
| 03W | NHS East Leicestershire and Rutland CCG | 0.69 |
| 09X | NHS Horsham and Mid Sussex CCG | 0.70 |
| 15A | NHS Berkshire West CCG | 0.71 |
| 10J | NHS North Hampshire CCG | 0.71 |
| 99F | NHS Castle Point and Rochford CCG | 0.72 |
| 09N | NHS Guildford and Waverley CCG | 0.72 |
| 99M | NHS North East Hampshire and Farnham CCG | 0.73 |
| 06Q | NHS Mid Essex CCG | 0.74 |
| 99J | NHS West Kent CCG | 0.74 |
| 11A | NHS West Hampshire CCG | 0.74 |
| 99D | NHS South Lincolnshire CCG | 0.75 |
| 08J | NHS Kingston CCG | 0.75 |
| 15D | NHS Berkshire East CCG | 0.75 |
| 06Y | NHS South Norfolk CCG | 0.76 |
| 09L | NHS East Surrey CCG | 0.77 |
| 06N | NHS Herts Valleys CCG | 0.77 |
| 08P | NHS Richmond CCG | 0.78 |
| 04Q | NHS South West Lincolnshire CCG | 0.78 |
| 07G | NHS Thurrock CCG | 0.78 |
| 05R | NHS South Warwickshire CCG | 0.78 |
| 07H | NHS West Essex CCG | 0.78 |
| 10K | NHS Fareham and Gosport CCG | 0.78 |
| 09H | NHS Crawley CCG | 0.78 |
| 08F | NHS Havering CCG | 0.78 |
| 03E | NHS Harrogate and Rural District CCG | 0.79 |
| 04V | NHS West Leicestershire CCG | 0.79 |
| 07K | NHS West Suffolk CCG | 0.79 |
| 03Q | NHS Vale of York CCG | 0.80 |
| 05V | NHS Stafford and Surrounds CCG | 0.80 |
| 99E | NHS Basildon and Brentwood CCG | 0.80 |
| 05D | NHS East Staffordshire CCG | 0.80 |
| 03D | NHS Hambleton, Richmondshire and Whitby CCG | 0.80 |
| 99N | NHS Wiltshire CCG | 0.80 |
| 06K | NHS East and North Hertfordshire CCG | 0.80 |
| 05Q | NHS South East Staffordshire and Seisdon Peninsula CCG | 0.80 |
| 01C | NHS Eastern Cheshire CCG | 0.81 |
| 07Q | NHS Bromley CCG | 0.81 |
| 07N | NHS Bexley CCG | 0.81 |
| **CCG code** | **CCG Name** | **Need Index** |
| 99K | NHS High Weald Lewes Havens CCG | 0.81 |
| 11E | NHS Bath and North East Somerset CCG | 0.81 |
| 04Y | NHS Cannock Chase CCG | 0.81 |
| 06F | NHS Bedfordshire CCG | 0.82 |
| 09C | NHS Ashford CCG | 0.82 |
| 05T | NHS South Worcestershire CCG | 0.82 |
| 10Q | NHS Oxfordshire CCG | 0.82 |
| 10V | NHS South Eastern Hampshire CCG | 0.82 |
| 09W | NHS Medway CCG | 0.83 |
| 05J | NHS Redditch and Bromsgrove CCG | 0.83 |
| 09J | NHS Dartford, Gravesham and Swanley CCG | 0.84 |
| 06L | NHS Ipswich and East Suffolk CCG | 0.84 |
| 04F | NHS Milton Keynes CCG | 0.85 |
| 05H | NHS Warwickshire North CCG | 0.85 |
| 08G | NHS Hillingdon CCG | 0.86 |
| 02Y | NHS East Riding of Yorkshire CCG | 0.86 |
| 08T | NHS Sutton CCG | 0.86 |
| 11M | NHS Gloucestershire CCG | 0.87 |
| 05F | NHS Herefordshire CCG | 0.87 |
| 04L | NHS Nottingham North and East CCG | 0.87 |
| 02D | NHS Vale Royal CCG | 0.88 |
| 08E | NHS Harrow CCG | 0.88 |
| 02A | NHS Trafford CCG | 0.88 |
| 04H | NHS Newark & Sherwood CCG | 0.88 |
| 05N | NHS Shropshire CCG | 0.88 |
| 08N | NHS Redbridge CCG | 0.88 |
| 06H | NHS Cambridgeshire and Peterborough CCG | 0.88 |
| 12D | NHS Swindon CCG | 0.89 |
| 04M | NHS Nottingham West CCG | 0.89 |
| 04G | NHS Nene CCG | 0.89 |
| 00X | NHS Chorley and South Ribble CCG | 0.89 |
| 01R | NHS South Cheshire CCG | 0.89 |
| 06V | NHS North Norfolk CCG | 0.90 |
| 06D | NHS Wyre Forest CCG | 0.90 |
| 03K | NHS North Lincolnshire CCG | 0.90 |
| 08R | NHS Merton CCG | 0.91 |
| 11X | NHS Somerset CCG | 0.91 |
| 10D | NHS Swale CCG | 0.92 |
| 02F | NHS West Cheshire CCG | 0.92 |
| 07M | NHS Barnet CCG | 0.93 |
| 01W | NHS Stockport CCG | 0.93 |
| 02G | NHS West Lancashire CCG | 0.93 |
| 02E | NHS Warrington CCG | 0.94 |

| **CCG code** | **CCG Name** | **Need Index** |
| --- | --- | --- |
| 09G | NHS Coastal West Sussex CCG | 0.94 |
| 05G | NHS North Staffordshire CCG | 0.95 |
| 11J | NHS Dorset CCG | 0.95 |
| 99P | NHS Northern, Eastern and Western Devon CCG | 0.95 |
| 07J | NHS West Norfolk CCG | 0.95 |
| 02N | NHS Airedale, Wharfedale and Craven CCG | 0.96 |
| 15C | NHS Bristol, North Somerset and South Gloucestershire CCG | 0.96 |
| 07Y | NHS Hounslow CCG | 0.96 |
| 02Q | NHS Bassetlaw CCG | 0.96 |
| 26A | NHS Derbyshire CCG | 0.97 |
| 04D | NHS Lincolnshire West CCG | 0.97 |
| 09E | NHS Canterbury and Coastal CCG | 0.98 |
| 03V | NHS Corby CCG | 0.98 |
| 11N | NHS Kernow CCG | 0.98 |
| 01E | NHS Greater Preston CCG | 0.98 |
| 05C | NHS Dudley CCG | 0.99 |
| 05A | NHS Coventry and Rugby CCG | 0.99 |
| 02M | NHS Fylde & Wyre CCG | 0.99 |
| 03T | NHS Lincolnshire East CCG | 0.99 |
| 00V | NHS Bury CCG | 0.99 |
| 10R | NHS Portsmouth CCG | 1.00 |
| 03J | NHS North Kirklees CCG | 1.00 |
| 06T | NHS North East Essex CCG | 1.00 |
| 05X | NHS Telford and Wrekin CCG | 1.00 |
| 00J | NHS North Durham CCG | 1.01 |
| 01H | NHS Cumbria CCG | 1.01 |
| 07L | NHS Barking and Dagenham CCG | 1.01 |
| 02H | NHS Wigan Borough CCG | 1.01 |
| 00Y | NHS Oldham CCG | 1.01 |
| 03L | NHS Rotherham CCG | 1.03 |
| 03A | NHS Greater Huddersfield CCG | 1.04 |
| 02T | NHS Calderdale CCG | 1.04 |
| 00L | NHS Northumberland CCG | 1.04 |
| 03R | NHS Wakefield CCG | 1.04 |
| 02X | NHS Doncaster CCG | 1.04 |
| 10A | NHS South Kent Coast CCG | 1.04 |
| 00T | NHS Bolton CCG | 1.05 |
| 09F | NHS Eastbourne, Hailsham and Seaford CCG | 1.05 |
| 05Y | NHS Walsall CCG | 1.05 |
| 04E | NHS Mansfield and Ashfield CCG | 1.05 |
| 06P | NHS Luton CCG | 1.05 |
| 06M | NHS Great Yarmouth and Waveney CCG | 1.05 |
| 03H | NHS North East Lincolnshire CCG | 1.05 |

| **CCG code** | **CCG Name** | **Need Index** |
| --- | --- | --- |
| 01V | NHS Southport and Formby CCG | 1.06 |
| 01K | NHS Morecambe Bay CCG | 1.06 |
| 99C | NHS North Tyneside CCG | 1.06 |
| 03M | NHS Scarborough and Ryedale CCG | 1.07 |
| 01Y | NHS Tameside and Glossop CCG | 1.07 |
| 10L | NHS Isle of Wight CCG | 1.07 |
| 99Q | NHS South Devon and Torbay CCG | 1.07 |
| 06W | NHS Norwich CCG | 1.07 |
| 10X | NHS Southampton CCG | 1.08 |
| 02P | NHS Barnsley CCG | 1.08 |
| 03N | NHS Sheffield CCG | 1.08 |
| 07X | NHS Enfield CCG | 1.09 |
| 02R | NHS Bradford Districts CCG | 1.09 |
| 08X | NHS Wandsworth CCG | 1.10 |
| 01A | NHS East Lancashire CCG | 1.10 |
| 15F | NHS Leeds CCG | 1.10 |
| 00K | NHS Hartlepool and Stockton-on-Tees CCG | 1.10 |
| 01D | NHS Heywood, Middleton and Rochdale CCG | 1.11 |
| 04C | NHS Leicester City CCG | 1.12 |
| 00C | NHS Darlington CCG | 1.12 |
| 00P | NHS Sunderland CCG | 1.13 |
| 01F | NHS Halton CCG | 1.13 |
| 99G | NHS Southend CCG | 1.14 |
| 07W | NHS Ealing CCG | 1.16 |
| 15E | NHS Birmingham and Solihull CCG | 1.17 |
| 01X | NHS St Helens CCG | 1.18 |
| 08A | NHS Greenwich CCG | 1.18 |
| 05W | NHS Stoke on Trent CCG | 1.18 |
| 01T | NHS South Sefton CCG | 1.19 |
| 13T | NHS Newcastle Gateshead CCG | 1.20 |
| 00Q | NHS Blackburn with Darwen CCG | 1.20 |
| 12F | NHS Wirral CCG | 1.21 |
| 07V | NHS Croydon CCG | 1.21 |
| 09D | NHS Brighton and Hove CCG | 1.23 |
| 00D | NHS Durham Dales, Easington and Sedgefield CCG | 1.23 |
| 09P | NHS Hastings and Rother CCG | 1.23 |
| 02W | NHS Bradford City CCG | 1.23 |
| 03F | NHS Hull CCG | 1.23 |
| 08W | NHS Waltham Forest CCG | 1.24 |
| 01J | NHS Knowsley CCG | 1.24 |
| 08M | NHS Newham CCG | 1.24 |
| 06A | NHS Wolverhampton CCG | 1.25 |
| 04K | NHS Nottingham City CCG | 1.26 |

| **CCG code** | **CCG Name** | **Need Index** |
| --- | --- | --- |
| 10E | NHS Thanet CCG | 1.26 |
| 00N | NHS South Tyneside CCG | 1.27 |
| 01G | NHS Salford CCG | 1.27 |
| 00M | NHS South Tees CCG | 1.27 |
| 08V | NHS Tower Hamlets CCG | 1.28 |
| 07P | NHS Brent CCG | 1.31 |
| 14L | NHS Manchester CCG | 1.33 |
| 08D | NHS Haringey CCG | 1.34 |
| 05L | NHS Sandwell and West Birmingham CCG | 1.37 |
| 09A | NHS Central London (Westminster) CCG | 1.39 |
| 08C | NHS Hammersmith and Fulham CCG | 1.40 |
| 07R | NHS Camden CCG | 1.41 |
| 99A | NHS Liverpool CCG | 1.46 |
| 08Y | NHS West London CCG | 1.48 |
| 08L | NHS Lewisham CCG | 1.52 |
| 00R | NHS Blackpool CCG | 1.53 |
| 07T | NHS City and Hackney CCG | 1.56 |
| 08K | NHS Lambeth CCG | 1.56 |
| 08H | NHS Islington CCG | 1.62 |
| 08Q | NHS Southwark CCG | 1.62 |

1. Pay band 2 (Job role: 1010 or 1011or 7012 or 7015); Pay band 3 (Job role: 7000, 7001, 7002, 7004, 7027, 8000, 8001, 8002, 8003, 9001; Occupation code: N9E, H2, H1D, G2D, S4C); Pay band 4 (Job role: 2000, 2001, 2003, 2004, 2007, 2010, 2012, 2016, 2018, 2022, 2023, 7005, 7009, 7010, 7019, 7023, 7029, 7031, 8003, 8004, 8005, 8006; Occupation code: H2D, S4, N1B, S5U, S5L); Pay band 5 (Job role: 1023, 3000, 3007, 3008, 3015, 5000, 5011, 5012, 7028; Occupation code: N6, NAD, N9D, S8M, S1B; Specialty: 900, 950, 700); Pay band 6 (Job role: 1009, 3014, 4000, 4001, 4009, 4025, 4041, 4057, 5003, 5007, 5008, 5009, 5010, 5015, 5021, 7011, 8007; Occupation code: N7E, S1, N6E, N4, N0, S1U, N4D, G2A, N6F, N6D, NAE, N7D; Specialty: 960, 901, 710, 725, 727; Job role, occupation code and specialty: missing); Pay band 7 (Job role: 1004, 1024, 3003, 3005, 3006, 4003, 4004, 4007, 4012, 4013, 4015, 4016, 4017, 4018, 4019, 4021, 4027, 4028, 4042, 4044, 4059, 4060, 5004, 5014, 5016; Occupation code: S1C, S8L, SAM, N7, S1J, S1H, S1M, N0D, G0A, N60, NCE, S4L, S9J, G2, S1L, S3L; Specialty: 713); Pay band 8 or 9 (Job role: 1000, 1006, 1013, 1022, 3002, 3004, 5005, 8009, 1001, 1002, 1005, 1012, 1016, 1017, 1018, 1019, 1032, 1035, 1037; Occupation code: S0C, S2L, G2C, S0L, SAL, N0E, S0M, S2M). [↑](#footnote-ref-1)
